# Statement of Retraction: MicroRNA-139-5p acts as a suppressor gene for depression by targeting nuclear receptor subfamily 3, group C, member 1

**DOI:** 10.1080/21655979.2026.2728885

**Published:** 2026-10-01

We, the Editors and Publisher of the journal *Bioengineered*, have retracted the following article from publication.

Su, B., Cheng, S., Wang, L., & Wang, B. (2022). MicroRNA-139-5p acts as a suppressor gene for depression by targeting nuclear receptor subfamily 3, group C, member 1. *Bioengineered*, 13(5), 11856–11866. https://doi.org/10.1080/21655979.2022.2059937

Since publication, significant concerns have been raised by a third party about the integrity of the data and reported results in the article. Specifically, concerns were raised about alleged image duplication with a similar image from an unrelated article:

- Figure 3b second panel, appears to have been duplicated with a similar image in Figure 4A miR-383 inhibitor panel from Liu et al, 2021 (https://doi.org/10.1038/s41598-021-88560-6) 

When approached for an explanation, the authors did not respond to our queries and so these serious concerns remain unaddressed.

As verifying the validity of published work is core to the integrity of the scholarly record, we are therefore retracting the article. The authors have been informed of this decision.

We have been informed in our decision-making by our policy on publishing ethics and integrity and COPE guidelines. 

The retracted article will remain online to maintain the scholarly record and will be digitally watermarked on each page as ‘Retracted’. 

